# Radiomic Signatures for Predicting Receptor Status in Breast Cancer Brain Metastases

**DOI:** 10.3389/fonc.2022.878388

**Published:** 2022-06-06

**Authors:** Xiao Luo, Hui Xie, Yadi Yang, Cheng Zhang, Yijun Zhang, Yue Li, Qiuxia Yang, Deling Wang, Yingwei Luo, Zhijun Mai, Chuanmiao Xie, Shaohan Yin

**Affiliations:** ^1^ State Key Laboratory of Oncology in South China, Collaborative Innovation Center for Cancer Medicine, Sun Yat-sen University Cancer Center, Guangzhou, China; ^2^ Department of Radiology, Sun Yat-Sen University Cancer Center, Guangzhou, China; ^3^ Department of Pathology, Sun Yat-Sen University Cancer Center, Guangzhou, China; ^4^ Department of Molecular Diagnostics, Sun Yat-Sen University Cancer Center, Guangzhou, China

**Keywords:** breast neoplasms, receptor, brain neoplasms, radiomics, magnetic resonance imaging

## Abstract

**Backgrounds:**

A significant proportion of breast cancer patients showed receptor discordance between primary cancers and breast cancer brain metastases (BCBM), which significantly affected therapeutic decision-making. But it was not always feasible to obtain BCBM tissues. The aim of the present study was to analyze the receptor status of primary breast cancer and matched brain metastases and establish radiomic signatures to predict the receptor status of BCBM.

**Methods:**

The receptor status of 80 matched primary breast cancers and resected brain metastases were retrospectively analyzed. Radiomic features were extracted using preoperative brain MRI (contrast-enhanced T1-weighted imaging, T2-weighted imaging, T2 fluid-attenuated inversion recovery, and combinations of these sequences) collected from 68 patients (45 and 23 for training and test sets, respectively) with BCBM excision. Using least absolute shrinkage selection operator and logistic regression model, the machine learning-based radiomic signatures were constructed to predict the estrogen receptor (ER), progesterone receptor (PR), and human epidermal growth factor receptor 2 (HER2) status of BCBM.

**Results:**

Discordance between the primary cancer and BCBM was found in 51.3% of patients, with 27.5%, 27.5%, and 5.0% discordance for ER, PR, and HER2, respectively. Loss of receptor expression was more common (33.8%) than gain (18.8%). The radiomic signatures built using combination sequences had the best performance in the training and test sets. The combination model yielded AUCs of 0.89, 0.88, and 0.87, classification sensitivities of 71.4%, 90%, and 87.5%, specificities of 81.2%, 76.9%, and 71.4%, and accuracies of 78.3%, 82.6%, and 82.6% for ER, PR, and HER2, respectively, in the test set.

**Conclusions:**

Receptor conversion in BCBM was common, and radiomic signatures show potential for noninvasively predicting BCBM receptor status.

## Introduction

Breast cancer is the most prevalent cancer worldwide (1) and the second-most likely solid malignancy to spread to the brain (2). Breast cancer produces highly heterogeneous tumors that are classified into clinically relevant subtypes based on the status of the estrogen receptor (ER), progesterone receptor (PR), human epidermal growth factor receptor 2 (HER2) and Ki67. Discordance in receptor status between primary breast tumors and metastatic disease has been increasingly reported (3–9). Such transformation can significantly impact treatment strategies, responses to therapy, and patient outcomes (6, 8, 10–14). Growing evidence suggests that it is good clinical practice to biopsy distant metastases to assess receptor status whenever possible; such assessments are recommended in American Society of Clinical Oncology and the joint European Association of Neuro-Oncology − European Society for Medical Oncology guidelines (15, 16). Clinical data have shown that the incidence of breast cancer brain metastases (BCBM) is increasing due to advances in systemic therapy and central nervous system imaging (2). In patients with extracranial disease that is under effective control, the development of new-onset or progressive brain metastases poses a clinical challenge due to the difficulties in identifying BCBM genetic status or receptor expression. Radiologists can depict the distribution, number, size, and morphological characteristics of brain metastases using MRI but cannot confirm the molecular alterations. Obtaining BCBM materials by biopsy or resection may not be practical or feasible depending on the patient’s performance status. Additionally, the risks of neurosurgery, sampling bias, and the fact that the procedure does not always provide an accurate account of the intrinsic intertumor and intratumor heterogeneity must be considered (3, 5, 9).These issues emphasize the need to develop an innovative approach for deriving metastasis biomarkers. Radiomics is an emerging technology that extracts high-dimensional features from images to mine the potential biological characteristics of tumors (17). Although several studies have applied radiomics to predict epidermal growth factor receptor (EGFR) or B-Raf proto-oncogene (BRAF) mutations in brain metastases (18–22), radiomic signatures associated with BCBM receptor status have not been reported.

Therefore, this study aimed to investigate receptor status in primary breast cancer and paired resected brain metastases and establish radiomic signatures to predict the ER, PR, and HER2 status of BCBM using preoperative brain MRI. We hypothesized that differential receptor expression between primary breast cancers and their brain metastases could be captured by radiomic signatures.

## Materials and methods

### Patients

**Figure 1:**
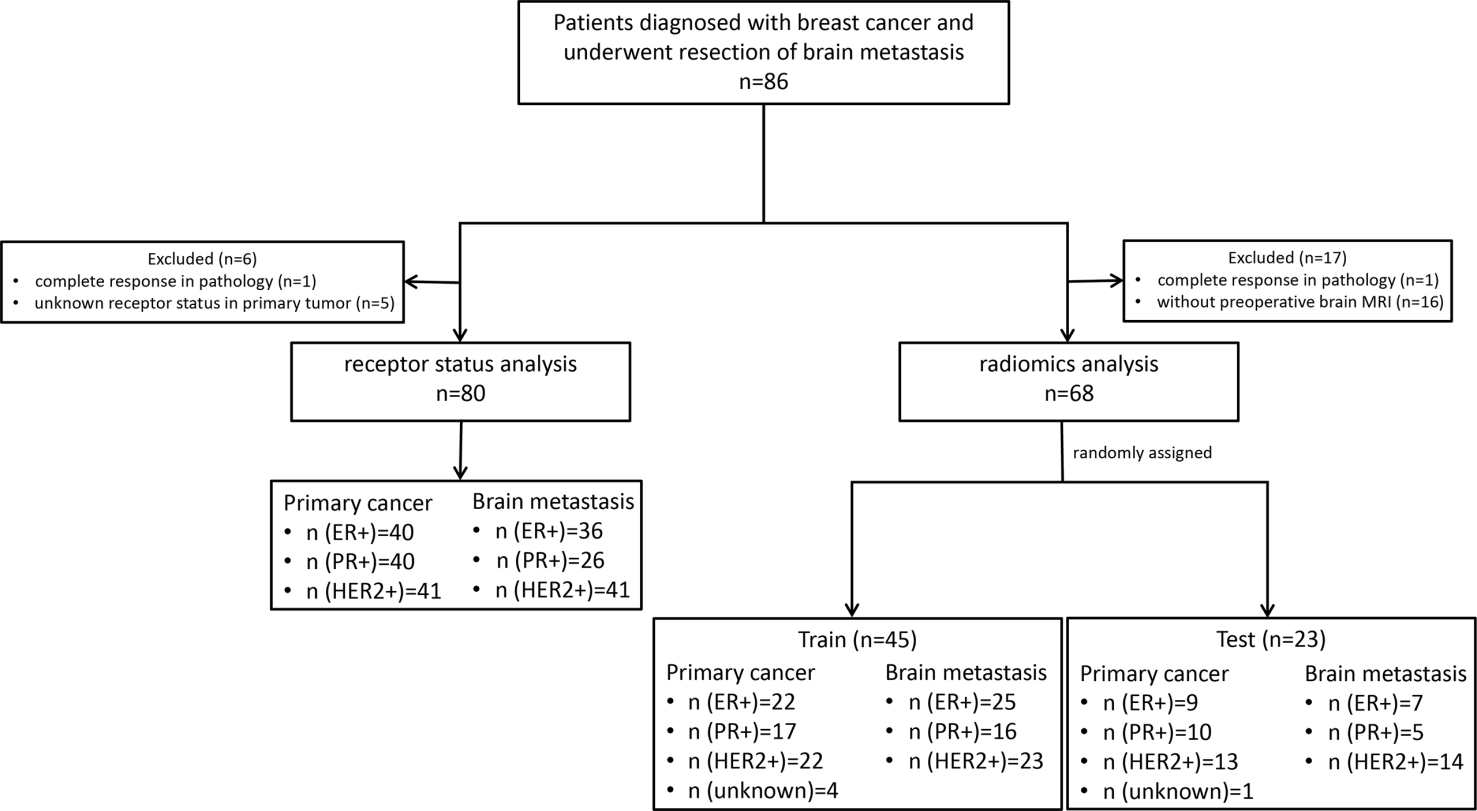
The flowchart of participants. ER, estrogen receptor; P, progesterone receptor; HER2, human epidermal growth factor receptor 2.

This retrospective single-center analysis included patients with breast cancer who consecutively underwent brain metastasis surgical resection at the Sun Yat-sen University Cancer Center between July 12, 2013 and September 19, 2021. The inclusion criteria were patients who: (a) had a primary breast tumor confirmed by biopsy or postoperative pathology; (b) had been diagnosed with BCBM; and (c) underwent brain metastasis surgical resection. For the receptor analysis, patients who did not have complete pathology data for the matched primary breast tumor and brain metastasis were excluded. For the radiomic analysis, patients who did not have complete pathology data for the brain metastasis and brain MRI were excluded (**Figure 1**). There were no limitations on patient gender and age. Clinical data were acquired from electronic medical records. Patients who were eventually enrolled were randomly assigned to the training and test sets (2:1), and there was no overlap patient between two sets.

This study was approved by the institutional review boards (No. B2021-198-01) of our center, and informed consent was exempted.

### ER, PR, and HER2 Status

Given that treatment selection can induce changes in receptor expression (6, 13), the ER, PR, and HER2 status of the primary tumor was determined from the pathology results after surgery for patients who did not receive neoadjuvant therapy. Puncture results were analyzed for patients who received neoadjuvant therapy or did not undergo surgery. Brain metastasis receptor status was assessed using surgical histopathology. ER and PR positive were defined as > 1% of tumor cell nuclei staining positively with any intensity. The histology and immunohistochemistry status of the breast cancer and matched metastases were analyzed by a pathologist with 8 years of experience according to the World Health Organization criteria (23). HER2 positive was defined as HER2 membrane staining score 3+ by immunohistochemistry or 2+ with fluorescence *in-situ* hybridization or HER2 amplification interpreted *via* next-generation sequencing technology by a molecular diagnostician with 4 years of experience. Hormone receptor (HR) status positive was defined as ER or PR positive.

### Image Acquisition

Sixty-eight eligible patients underwent brain MRI with 1.5-T (8 patients) or 3.0-T (60 patients) scanners. Contrast-enhanced T1-weighted imaging (T1CE), T2-weighted imaging (T2WI) and T2 fluid-attenuated inversion recovery (T2-FLAIR) were collected for feature extraction. The imaging parameters are provided in the **Supplementary Materials**. The MRI examination closest to surgery was selected. For patients with multiple brain metastases, only the lesions matched with the surgical pathology were included in the radiomic analysis.

### Image Segmentation and Radiomic Feature Extraction and Selection

**Figure 2:**
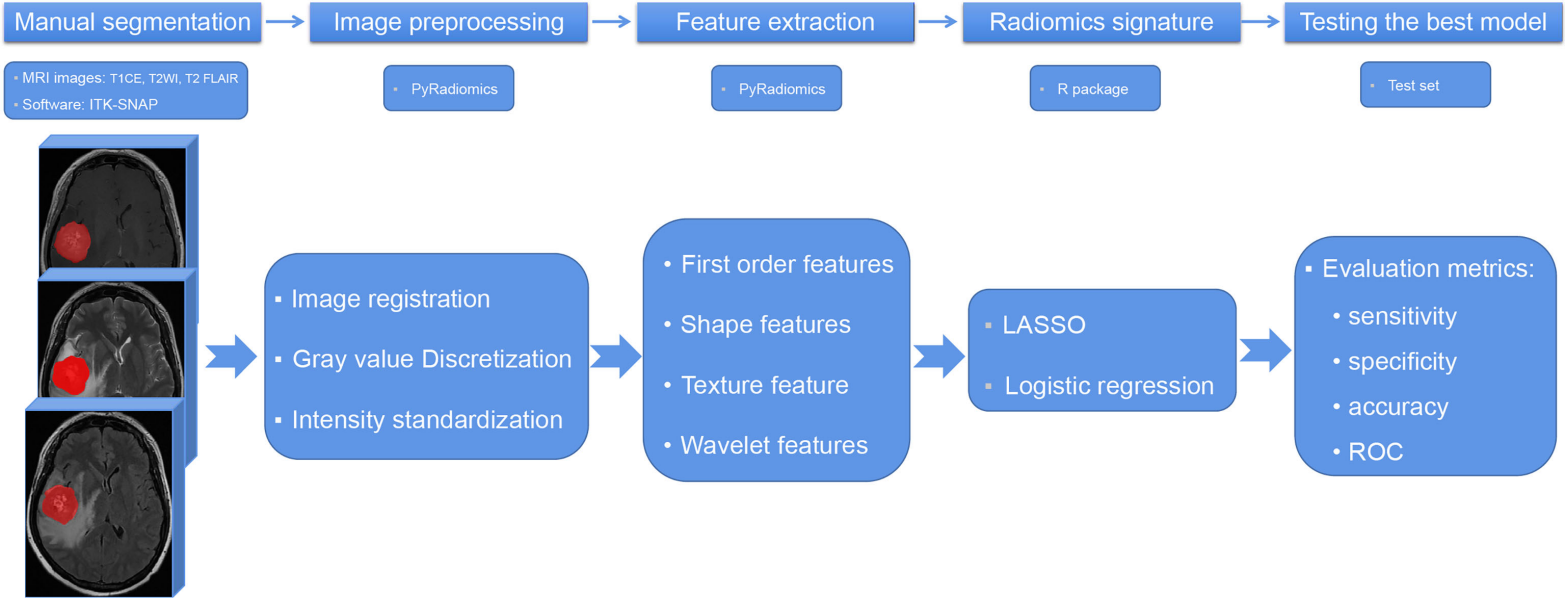
The flowchart of radiomic analysis. T1CE, contrast-enhanced T1-weighted imaging; T2WI, T2-weighted imaging; T2 FLAIR, T2 fluid-attenuated inversion recovery; LASSO, least absolute shrinkage selection operator; ROC, receiver operating characteristic curve.

The radiomic analysis was processed as shown in **Figure 2**. Paired brain metastases imaged in the above three sequences were manually contoured around the lesions on the axial view by a junior radiologist with four years of experience using ITK-SNAP (version 3.6; www.itksnap.org). The region of interest avoided hemorrhagic, edematous, necrotic, and cystic areas. These segmentations were reviewed by a senior neuroradiologist with 12 years of experience and refined if necessary.

Radiomic features were extracted using PyRadiomics, an open-source Python package for the extraction of radiomic features from medical images (http://www.radiomics.io/pyradiomics.html). This radiomic quantification platform enables the standardization of both image processing and feature definitions (24). The gray value discretization was conducted with a fixed bin width of 25. Because MRI scanners with different field strengths were used, the intensity range of the images was normalized between 0 and 100 as a default set by the platform. We performed resampling with a pixel spacing of (3, 3, 3). The descriptions and feature explanations can be found on the PyRadiomics website. The parameter settings for feature extraction and image preprocessing details are provided as a.py file and a.yaml file in the **Supplementary Materials**.

The interclass correlation coefficient (ICC) was used to assess the stability of each feature. Ten patients were randomly selected from the cohort and segmented again by the same radiologist for the stability evaluation. Intraobserver stability was calculated for each feature (**Supplementary Figure 1**). Stable radiomic features were defined as ICCs > 0.9. An initial selection was performed by deleting collinear strongly correlated variables detected using Pearson’s correlation, for which the cutoff value was 0.95. A univariate analysis was performed for each feature, and features with P < 0.05 were considered for selection. Marginally significant features were selected using the least absolute shrinkage and selection operator (LASSO) and logistic regression model, which performed variable selection and regularization to enhance the prediction accuracy and interpretability of the statistical model. All features with non-zero coefficients were selected in this step. Finally, backward elimination was selectively performed to reduce the number of features included in the final set (**Supplementary Table 1**).

The radiomic model performance was internally tested using an independent test cohort. The discrimination performance of the established model was quantified using the receiver operating characteristic curve (ROC) and the area under the curve (AUC).

### Statistical Analysis

Statistical analyses were performed using R software version 4.0.2 (http://www.r-project.org/). The frequency of receptor expression in the primary cancers and BCBM was calculated and compared using McNemar’s test. Percentages of conversion were calculated for the whole receptors, and for each receptor. We used the following R packages: irr (version 0.84.1) for calculating ICCs; caret (version 6.0–86) for Pearson’s correlation analyses; glmnet (version 4.0–2) for LASSO logistic regression; rms (version 6.0–1) for logistic regression; and pROC (version 1.17) for ROC and AUC. The classification performance of the radiomic model was evaluated by the AUC, sensitivity, specificity, and accuracy. All statistical tests were two-sided, and *P* < 0.05 was considered statistically significant.

## Results

### Patient Characteristics

As shown in **Figure 1**, 86 patients with BCBM were enrolled. Six patients were excluded due to complete response revealed by postoperative pathology (n = 1) or unknown primary breast cancer receptor status (n = 5). Eighty patients with matched primary tumor and brain metastases were included in the receptor conversion analysis. For the radiomic feature extraction, 18 patients were excluded due to complete response (n = 1) or lacking preoperative brain MRI (n = 17). Thus, 68 patients were included in the BCBM receptor status prediction. The mean interval between MRI scanning and resection was 13.5 days (range, 3–34 days).

All patients were women with unilateral breast cancer who underwent a single metastasis excision. The mean age at the initial breast cancer diagnosis was 44 ± 9 years (range, 23–63 years) in both the receptor and radiomic analyses. Of the known primary tumor types, most (> 95%) were invasive ductal carcinoma (**Table 1**).

**Table 1:** Study patient characteristics.

Characteristics	Receptor status analysis	Radiomics analysis		
		Training	Test	
Number of Patients	80	45	23
Age ^a^ (mean ± SD, years)	44 ± 9	44 ± 9	43 ± 9
Primary tumor grade (n, %)			
IDC I	3 (3.8)	1 (2.2)	1 (4.3)
IDC II	25 (31.3)	12 (26.7)	9 (39.1)
IDC III	28 (35.0)	18 (40.0)	5 (21.7)
Special type	3 (3.8)	1 (2.2)^b^	1 (4.3)^c^
Unknown	20 (25.0)	1 (2.2)	7 (30.4)
Interval between the MRI and the BCBM resection (mean ± SD, days)	NA	15 ± 7	11 ± 7
Excised brain metastases			
Size ^d^ (mean ± SD, mm)	40 ± 13	40 ± 13	45 ± 13
Location (cerebrum, n, %)	56 (70.0)	31 (68.9)	19 (82.6)
Breast cancer family history			
Yes	0	0	0
No	80 (100)	45 (100)	23 (100)
Menopausal status ^e^			
Premenopausal	67 (83.8)	37 (82.2)	19 (82.6)
Postmenopausal	12 (16.2)	7 (15.6)	4 (17.3)

^a^at initial diagnosis of breast cancer; ^b^mucinous carcinoma; ^c^metaplastic carcinoma; ^d^maximum diameter at axial section; ^e^a patient underwent hysterectomy before breast cancer diagnosis included in the receptor status analysis and training group; SD, standard deviation; IDC, invasive ductal carcinoma; MRI, magnetic resonance imaging; BCBM, breast cancer brain metastases; NA, not applicable.

### Receptor Status

**Figure 3:**
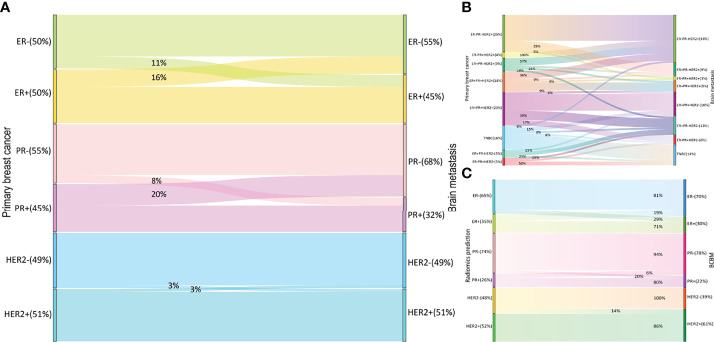
Receptor switch in BCBM and radiomics predicting receptor status in the test set. Receptor **(A)** and subtype **(B)** switch in BCBM; the prediction results for BCBM **(C)** BCBM, breast cancer brain metastases; ER, estrogen receptor; PR, progesterone receptor; HER2, human epidermal growth factor receptor 2.

The ER, PR, and HER2 conversion rates are summarized in **Figure 3A**. Among 80 paired samples, 50% (40/80), 45% (36/80), and 51% (41/80) of patients had ER-positive, PR-positive, and HER2-positive primary tumors, respectively, whereas in the corresponding BCBM these values were 45% (36/80), 33% (26/80), and 51% (41/80). The overall discordance between the primary cancer and the metastases was 51.3% (41/80), with conversion rates of 27.5% (22/80) for ER, 27.5% (22/80) for PR, and 5% (4/80) for HER2. HER2 was less likely to show discordance than ER or PR (both odds ratio [OR] = 0.139, 95% confidence interval [CI]: 0.045–0.425). The conversion from positive to negative (33.8%, 27/80) occurred significantly more often than from negative to positive (18.8%, 15/80) (OR = 2.208, 95% CI: 1.066–4.572). Patients with PR-positive had a higher rate of receptor discordance than patients with PR-negative (44.4% vs 13.6%, OR = 5.067, 95% CI: 1.715–14.969). A similar trend was seen for ER conversion, but the difference was not statistically significant (32.5% vs 22.5%, OR = 1.658, 95% CI: 0.614–4.482). No significant difference in discordance was detected between patients with HER2 positive and negative (4.9% vs 5.1%, OR = 0.949, 95% CI: 0.127–7.087). Subtype changes between the primary breast cancer and BCBM are illustrated in **Figure 3B**. The HR-negative/HER2-positive subtype was the most common in both primary tumors (25%, 20/80) and BCBM (33%, 26/80). The total subtype discordance was 51% (41/80). Of the discordant cases, higher conversion rates were observed in patients with HR-negative/HER2-positive (6%, 5/80), HR-positive/HER2-negative (6%, 5/80), and triple-positive (5%, 4/80).

### Feature Selection and Radiomic Signature Construction

For each MRI sequence and receptor, we built radiomic signatures using the training set and evaluated their classification performance in the test set. We extracted 1,470 radiomic features from each sequence, comprising 14 shape features, 288 first-order features, 352 gray-level co-occurrence matrix features, 224 gray-level dependence matrix features, 256 gray-level run-length matrix features, 256 gray-level size-zone matrix features, and 80 neighboring gray-tone difference matrix features (**Supplementary Table 1**).

The number of radiomic features selected to differentiate the ER, PR, and HER2 status was reduced to nine, eight, and six, respectively, from the combination sequences to build the radiomic model. **Table 2** lists the significant features for differentiating receptor status in the combination sequence model. Most selected features for the ER and PR were from T2 FLAIR (5/9 and 3/6), and most features for HER2 were from T2WI (6/8).

**Table 2:** Radiomic features to differentiate receptor status in combination model.

Receptor	Sequence	Feature category	Features
ER			
	T1CE	NGTDM	Busyness
	T1CE	GLDM	Dependence variance
	T2WI	GLSZM	Small area low gray level emphasis
	T2WI	First-order statistics	Maximum
	T2 FLAIR	GLCM	Cluster prominence
	T2 FLAIR	GLCM	Inverse variance
	T2 FLAIR	GLCM	Informational measure of correlation 1
	T2 FLAIR	GLRLM	Long run high gray level emphasis
	T2 FLAIR	GLCM	Cluster shade
PR			
	T1CE	GLDM	Dependence non uniformity normalized
	T2WI	GLCM	Informational measure of correlation 1
	T2WI	NGTDM	Contrast
	T2WI	GLDM	Dependence variance
	T2WI	GLSZM	Low gray level zone emphasis
	T2WI	GLRLM	Run length non uniformity
	T2WI	GLDM	Dependence variance
	T2 FLAIR	GLCM	Informational measure of correlation 1
HER2			
	T1CE	GLDM	Large dependence high gray level emphasis
	T1CE	First-order statistics	Skewness
	T2WI	GLSZM	Zone variance
	T2 FLAIR	GLCM	Inverse variance
	T2 FLAIR	First-order statistics	Mean
	T2 FLAIR	GLDM	Dependence variance

T1CE, contrast-enhanced T1-weighted imaging; T2-FLAIR, T2 fluid-attenuated inversion recovery; T2WI, T2-weighted imaging; ER, estrogen receptor, PR, progesterone receptor, HER2, human epidermal growth factor receptor 2; NGTDM, neighboring gray tone difference matrix; GLDM, gray level dependence matrix; GLSZM, gray level size zone matrix; GLCM, gray level co-occurrence matrix; GLRLM, gray level run length matrix.

### Prediction Performance

**Figure 4:**
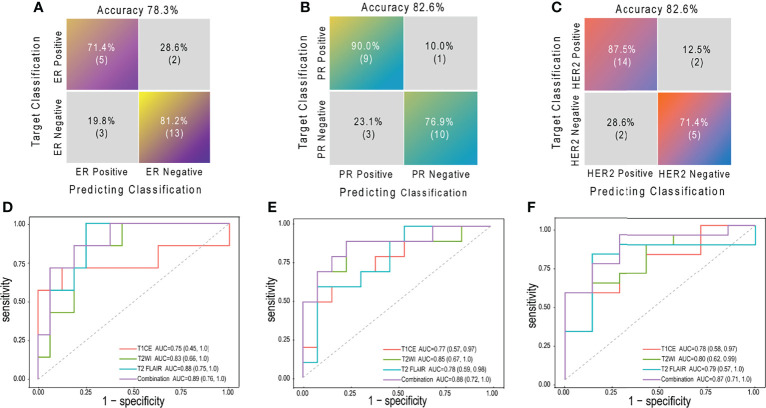
The confusion matrices and ROCs of combination radiomic signatures in test set. Confusion matrices for ER **(A)**, PR **(B)** and HER2 **(C)**; ROCs for ER **(D)**, PR **(E)** and HER2 **(F)** ROC, receiver operating characteristic curve; ER, estrogen receptor; PR, progesterone receptor; HER2, human epidermal growth factor receptor 2; AUC, area under the curve; T1CE, contrast-enhanced T1-weighted imaging; T2WI, T2-weighted imaging; T2 FLAIR, T2 fluid-attenuated inversion recovery; combination, combination features of three sequences above.

Prediction performance details are provided in **Table 3** and **Figures 3C**, **4**. Overall, the combination sequences achieved the best AUC for each receptor in the training and test sets, with AUCs of 0.89, 0.88, and 0.87, classification sensitivities of 71.4%, 90%, and 87.5%, specificities of 81.2%, 76.9%, and 71.4%, and accuracies of 78.3%, 82.6%, and 82.6% in the test set for ER, PR, and HER2, respectively. However, the AUCs were not significantly different between the combination sequences and the single sequences in the test (all *P* > 0.05).

**Table 3:** The radiomic performance of predicting receptor status in BCBM using different sequences.

	Training					Test				
Receptor	Sensitivity(%, 95% CI)	Specificity (%, 95% CI)	Accuracy(%, 95% CI)	AUC(95% CI)	*P* ^a^	Sensitivity(%, 95% CI)	Specificity(%, 95% CI)	Accuracy(%, 95% CI)	AUC(95% CI)	*P* ^a^
ER										
T1CE	84.0 (69.6, 98.4)	65.0 (44.1,85.9)	75.6 (74.8, 764)	0.76 (0.61, 0.90)	0.003*	71.4 (29.0,96.3)	62.5 (35.4, 84.8)	65.2 (42.7, 83.6)	0.75 (0.45, 1.0)	0.258
T2WI	84.0 (69.6, 98.4)	90.0 (76.9, 100.0)	86.7 (86.2, 87.2)	0.91 (0.83, 0.99)	0.133	100.0 (59.0, 100.0)	56.2 (29.9, 80.2)	69.6 (47.1, 86.8)	0.83 (0.66, 1.0)	0.398
T2 FLAIR	80.0 (64.3,95.7)	95.0 (85.4, 100.0)	86.7 (86.2, 87.2)	0.93 (0.85,1.0)	0.230	57.1 (18.4, 90.1)	93.80 (69.8, 99.8)	82.6 (61.2, 95.0)	0.88 (0.75, 1.0)	0.903
Combination	100.0 (100.0, 100.0)	90.0 (76.9, 1.00)	95.6 (95.4, 95.7)	0.96 (0.91, 1.0)		71.4 (29.0, 96.3)	81.2 (54.4, 96.0)	78.3 (56.3,92.5)	0.89 (0.76, 1.0)	
PR										
T1CE	81.8 (59.0, 100.0)	64.7 (48.6, 80.8)	68.9 (68.0, 69.8)	0.76 (0.60, 0.91)	0.036*	60.0 (26.2, 87.8)	76.9 (46.2, 95.0)	69.6 (47.1, 86.8)	0.77 (0.57, 0.97)	0.422
T2WI	90.9 (73.9, 100.0)	82.4 (69.5, 95.2)	84.4 (83.9, 85.0)	0.93 (0.85, 1.0)	0.850	70.0 (34.8, 93.3)	84.6 (54.6, 98.1)	78.3 (56.3, 92.5)	0.85 (0.67, 1.0)	0.259
T2 FLAIR	63.6 (35.2, 92.1)	85.3 (73.4, 97.2)	80.0 (79.3, 80.7)	0.75 (0.59, 0.91)	0.020*	40.0 (12.2, 73.8)	92.3 (64.0, 99.8)	69.6 (47.1, 86.8)	0.78 (0.59, 0.98)	0.444
Combination	100.0 (100.0, 100.0)	79.4 (65.8, 93.0)	84,4 (83.9, 85.0)	0.93 (0.86, 1.0)		90.0 (55.5, 99.7)	76.9 (46.2, 95.0)	82.6 (61.2, 95.0)	0.88 (0.72, 1.0)	
HER2										
T1CE	85.7 (70.7, 100.0)	66.7 (47.8,85.5)	75.6 (74.8, 76.4)	0.77 (0.63, 0.91)	0.014*	56.2 (29.9, 80.2)	71.4 (29.0, 96.3)	60.9 (38.5, 80.3)	0.78 (0.58, 0.97)	0.295
T2WI	66.7 (46.5, 86.8)	79.2 (62.9, 95.4)	73.3 (72.5, 74.2)	0.75 (0.61, 0.90)	0.008*	56.2 (29.9, 80.2)	100.0 (59.0, 100.0)	69.6 (47.1, 86.8)	0.80 (0.62, 0.99)	0.510
T2 FLAIR	100.0 (100.0, 100.0)	83.3 (68.4, 98.2)	91.1 (90.8, 91.5)	0.94 (0.86, 1.0)	0.563	87.5 (61.7, 98.4)	57.1 (18.4,90.1)	78.3 (56.3, 92.5)	0.79 (0.57, 1.0)	0.192
Combination	100.0 (100.0, 100.0)	87.5 (74.3, 100.0)	93.3 (93.1, 93.6)	0.96 (0.90, 1.0)		87.5 (61.7, 98.4)	71.4 (29.0, 96.3)	82.6 (61.2, 95.0)	0.87 (0.71, 1.0)	

^a^the AUC of T1CE, T2WI and T2 FLAIR compared with the combination of that three sequences, respectively; *, statistically significant; BCBM, breast cancer brain metastases; AUC, area under the curve; CI, confidence interval; ER, estrogen receptor; PR, progesterone receptor; HER2, human epidermal growth factor receptor 2; T1CE, contrast-enhanced T1-weighted imaging; T2-FLAIR, T2 fluid-attenuated inversion recovery; T2WI, T2-weighted imaging.

For 63 patients (41 and 22 in training and test sets, respectively) with available receptor status for matched primary breast cancer and BCBM, an overall conversion rate of 57% (36/63) was observed, with discordances of 27% (17/63) for ER, 27% (17/63) for PR, and 3% (2/63) for HER2. Overall, radiomic signatures achieved a BCBM classification accuracy of 85% in the test set (**Figure 3C**). The total discordance between breast cancer and the paired BCBM was 64% (14/22), with discordances of 32% (7/22) for ER, 25% (8/22) for PR, and 5% (1/22) for HER2. The overall classification accuracy of the radiomic model for discordant cases was 76% (11/14; 3 for ER, 7 for PR, and 1 for HER2).

## Discussion

In this retrospective study, we analyzed the ER, PR, and HER2 status of matched primary breast cancers and resected BCBM. The overall discordance rate between the primary cancer and the metastasis receptor status was 51.3%; the individual rates were 27.5% for ER, 27.5% for PR, and 5% for HER2. Conversion from positive to negative occurred more frequently than negative to positive, significantly so for PR. Given that this phenomenon may impact therapeutic decision-making and the barriers to BCBM material collection in clinical practice, we developed radiomic signatures based on preoperative brain MRI to predict the ER, PR, and HER2 status of BCBM. Integrative radiomic features predicted BCBM receptor status with AUCs of 0.89, 0.88, and 0.87 for ER, PR, and HER2, respectively. The integrative signatures correctly identified 76% of cases with discordance between the primary breast cancer and BCBM in the test set. Our findings support that breast cancer is a highly heterogeneous disease, highlighting the importance of reassessing BCBM receptor status to guide systemic therapy. The radiomics could potentially provide a noninvasive imaging biomarker for evaluating BCBM receptor phenotypes.

A recent meta-analysis detected a 42.6% overall receptor discordance between the primary breast cancer and BCBM, with 17.0% for ER, 23.0% for PR, and 12.0% for HER2 (25). Another systematic review reported a 22% total receptor discordance (9). The total conversion rate in this study was higher at 51.3%, but we found a lower HER2 discordance rate of 5%. Loss of receptor expression was more common (33.8%) than gain (18.84%), which was consistent with previous reports (3, 5, 13, 25). Breast cancer subtypes impact the BCBM incidence, kinetics, and prognosis (26); however, data on BCBM subtype switch are limited. Our analysis showed a tendency toward HR-negative/HER2-positive and ER-positive/PR-negative/HER2-negative subtypes and a trend away from the HR-positive/HER2-negative and triple-positive subtypes from the primary tumor to the BCBM (**Figure 3B**). These findings differ from Alexander et al. (9), in which the trend was toward triple-negative and HER2-positive subtypes and away from ER-positive/HER2-positive subtypes.

In the case of receptor loss, patients may suffer from therapy response failure at the cost of related toxicity. Alternatively, patients may miss an opportunity to receive effective treatments due to a lack of knowledge about receptor gain in metastases. Both circumstances could impact patient survival (13). Guidelines recommend retesting receptor status for metastases (15, 16); however, given the challenges in routinely obtaining intracranial tissue, BCBM are underrepresented. Minimally invasive techniques for evaluating circulating cell-free tumor DNA in the cerebrospinal fluid have been developed (27), but there is inadequate evidence supporting the utility of this technique as a reliable alternative to biopsies for determining BCBM receptor status.

Radiomic analysis enables noninvasive assessments of tumor status and relevant molecular information. Limited studies have reported promising results for differentiating brain metastasis molecular status using radiomics (19–22). Shofty et al. applied a machine-learning method to predict BRAF mutation in brain metastases using brain MRI in 53 patients with surgical resection from melanoma, achieving a mean accuracy of 79%, mean sensitivity of 72%, and AUC of 0.78 (20). However, the study did not include an independent test set to assess the performance, which could result in overfitting. A study evaluated EGFR mutation status in 99 brain metastases from 51 patients with lung cancer, resulting in an AUC, accuracy, sensitivity, and specificity of 0.73, 78.6%, 81.3%, and 76.9%, respectively (21). However, extracting features from multiple lesions within a patient could generate overlapping features. Another study by Wang et al. extracted features from T1CE, T2-FLAIR, T2WI and diffusion-weighted imaging (DWI) to extract features from 52 patients with lung adenocarcinoma (22). The radiomic signature of T2-FLAIR yielded an excellent AUC of 0.987, a classification accuracy of 99.1%, sensitivity of 100%, and specificity of 98.0% in the validation cohort. However, the EGFR mutation status in that study were evaluated in lung cancer tissues, which may result in inauthentic performance due to discordance between primary lung cancer and brain metastases, which is reportedly up to 26.5% (21, 28).

To our knowledge, radiomics for predicting BCBM receptor status has not been published yet. As we evaluated the receptor status in resected brain materials, our model may be more accurate than those deriving receptor status from primary cancers. We found that significant radiomic features selected from multiple sequences seemed to generate a superior AUC compared with single sequence, which is in line with Park et al. (21), who reported that features selected from the integration of T1CE and diffusion tensor images improved EGFR mutation status differentiation in brain metastases from lung cancer. For single sequence applied to predict ER and HER2 status, we found that the radiomic signature of T2-FLAIR had the best performance, consistent with Wang et al. (22), who found that T2-FLAIR yielded better EGFR mutation discrimination than TICE, T2WI, and DWI. For PR, radiomic signatures extracted from T2WI had the best performance. Our results indicate that single sequence have different predictive values for different receptors. Furthermore, more second-order features than first-order statistics were included, suggesting that multiparametric high-throughput characteristics enable a more accurate assessment.

There are several limitations to this study. First, this is a retrospective single-center design, which may create selection bias. The model performance should be validated using a larger prospective multi-center dataset. Nonetheless, this is a primary study to explore the feasibility of classifying BCBM receptor expression using radiomics. In patients with limited brain metastases, local therapy such as surgical resection or radiotherapy is the gold standard, but the systemic treatment is often continued (16, 29). Using our models, this could lead to a local therapy but also, in some patients, to a change in systemic therapies because of a modification of the receptor status. Besides, there are clinical reasons for the resection which could introduce a bias. Second, the sample size is not big enough because these samples are not easy to come by in clinical practice. Third, as the prediction performance of our model is not perfect, more novel techniques such as deep learning or functional MRI imaging should be investigated to extract features in future study. However, using an open-source Python package to extract features may improve reproducibility. In addition, conventional MRI sequences have wider adaptability in clinical practices. Due to the limitations of current radiomic technology, brain metastases tissue, obtained by biopsy or excision, is still necessary if it is practical and feasible. Third, we did not assess therapeutic regimen changes and their impact on patient outcomes because that was not within the study scope.

## Conclusion

In conclusion, receptor conversion was common in BCBM, and reappraising receptor status is necessary in clinical practice. Our multiparametric radiomic model can noninvasively predict the receptor status for BCBM, which will facilitate improved patient care and outcomes.

## Data Availability Statement

The original contributions presented in the study are included in the article/**Supplementary Material**. Further inquiries can be directed to the corresponding authors.

## Ethics Statement

The studies involving human participants were reviewed and approved by the institutional review board of Sun Yat-sen University Cancer Center. Written informed consent for participation was not required for this study in accordance with the national legislation and the institutional requirements.

## Author Contributions

XL, XHX, CX, and SY contributed to conception and design of the study. YY, YZ, YueL, QY, and DW organized the database. CZ, YingL, and ZM performed the statistical analysis. XL and HX wrote the first draft of the manuscript. YY and SY wrote sections of the manuscript. All authors contributed to manuscript revision, read, and approved the submitted version.

## Conflict of Interest

The authors declare that the research was conducted in the absence of any commercial or financial relationships that could be construed as a potential conflict of interest.

## Publisher’s Note

All claims expressed in this article are solely those of the authors and do not necessarily represent those of their affiliated organizations, or those of the publisher, the editors and the reviewers. Any product that may be evaluated in this article, or claim that may be made by its manufacturer, is not guaranteed or endorsed by the publisher.
