# Supplementary material for: Radiomic Signatures for Predicting Receptor Status in Breast Cancer Brain Metastases
Source: Front Oncol. 2022 Jun 6;12:878388. doi: 10.3389/fonc.2022.878388 (PMC9207517; doi:10.3389/fonc.2022.878388)
Supplement: Supplementary file 3 [file DataSheet_1.docx]

**Supplementary Materials**

1. **Three sequences imaging parameters in three scanners** (1.5-T Signa HDxt, GE Healthcare; 3.0-T Discovery MR750w, GE Healthcare; 3.0-T Prisma, Siemens)

T1-CE sequences were acquired 5 min after contrast injection (MultiHance; Bracco Diagnostics, Princeton, NJ), and the scanning parameters were as follows (Signa HDxt/Discovery MR750w/Prisma): repetition time (TR), 633/170/250 ms; echo time (TE), 9/1,928/2.46 ms; field of view (FOV), 240 mm × 240 mm; voxel size, 0.63 mm × 1.1 mm/0.63 mm × 0.94 mm/0.63 mm × 1.03 mm; matrix, 384 × 224/384 × 256/384 × 234 ; slice thickness, 5 mm; slice gap, 1 mm.

T2WI imaging parameters were as follows: TR, 5,266/11,565/5,150 ms; TE, 92/79.8/99 ms; FOV, 240 mm × 240 mm; voxel size, 0.67 mm × 1.1 mm/0.63 mm × 0.63 mm/0.63 mm × 0.63 mm; matrix, 512 × 224/384 × 384//384 × 384; slice thickness, 5 mm; slice gap, 1 mm.

T2-FLAIR imaging parameters were as follows: TR, 8002/9000/8000 ms; TE, 126/89.5/98 ms; FOV, 240 mm × 240 mm; voxel size, 0.86 mm × 1.26 mm/0.75 mm × 0.94 mm/0.63 mm × 0.78 mm; matrix, 280 × 190/320 × 256/384 × 307; slice thickness, 5 mm; slice gap, 1 mm.

1. **Supplementary figure legend**

**Figure S1 Interclass correlation coefficient for different sequences**

ICC=interclass correlation coefficient, glcm=gray level co-occurrence matrix, glrlm=gray level run length matrix, glszm=gray level size zone matrix, gldm=gray level dependence matrix, ngtdm=neighbouring gray tone difference matrix

**Figure S2 Decision curve analysis of ER, PR and HER2**

Decision curve analysis of ER (a), PR (b) and HER2 (c) in the test set; ER=estrogen receptor, PR=progesterone receptor, HER2=human epidermal growth factor receptor 2

1. **Supplementary table**

Table 1 Features extracted and selected for receptor status radiomic signatures

| Receptor |  | MRI sequence | | | |
| --- | --- | --- | --- | --- | --- |
|  |  | T1CE | T2WI | T2-FLAIR | Combination^a^ |
| ER |  |  |  |  |  |
| Total features extracted |  | 1,470 | 1,470 | 1,470 |  |
| ICC |  | 1,284 | 1,201 | 1,298 |  |
| Pearson correlation |  | 527 | 495 | 497 |  |
| Univariate analysis |  | 23 | 82 | 39 |  |
| LASSO |  | 4 | 9 | 16 |  |
| Backward elimination |  | NA | 5 | 6 | 9 |
| PR |  |  |  |  |  |
| Total features extracted |  | 1,470 | 1,470 | 1,470 |  |
| ICC |  | 1,284 | 1,201 | 1,298 |  |
| Pearson correlation |  | 527 | 495 | 497 |  |
| Univariate analysis |  | 20 | 88 | 64 |  |
| LASSO |  | 3 | 10 | 8 |  |
| Backward elimination |  | NA | 6 | 3 | 8 |
| HER2 |  |  |  |  |  |
| Total features extracted |  | 1,470 | 1,470 | 1,470 |  |
| ICC |  | 1,284 | 1,201 | 1,298 |  |
| Pearson correlation |  | 527 | 495 | 497 |  |
| Univariate analysis |  | 12 | 8 | 28 |  |
| LASSO |  | 7 | 4 | 12 |  |
| Backward elimination |  | NA | 3 | 6 | 6 |

a, combination of T1CE, T2WI and T2-FLAIR, T1CE=contrast-enhanced T1-weighted imaging, T2-FLAIR=T2 fluid-attenuated inversion recovery, T2WI=T2-weighted imaging, ER=estrogen receptor, PR=progesterone receptor, HER2=human epidermal growth factor receptor 2, ICC=interclass correlation coefficient, LASSO=least absolute shrinkage and selection operator, NA=no available feature
